# Use of bone scintigraphy in the early diagnosis of bisphosphonate related osteonecrosis of the jaw. Case report and review of the literature

**DOI:** 10.4317/jced.55248

**Published:** 2018-12-01

**Authors:** José-Darío Sánchez-López, Paolo Cariati, Jacobo Cambil-Martin, Mercedes Villegas-Calvo, María-Luisa Moreno-Martin

**Affiliations:** 1 Department of Oral an Maxillofacial Surgery. Universitary Hospital “Virgen de las Nieves”. Granada (Spain); 2 Department of Healthcare . University of Granada (Spain); 3 Department of Surgery. Universitary Hospital “Virgen de las Nieves”. Granada (Spain); 4 Department of Anestesiology. Universitary Hospital “Virgen de las Nieves”. Granada (Spain)

## Abstract

The main aim of the present report is to show the potential utility of bone scintigraphy for the diagnosis of jaw osteonecrosis. We report the history of a 62-year-old woman underwent breast cancer surgery in 2010. Moreover, patient received postoperative radiotherapy and chemotherapy. Intravenous bisphosphonates were also added to the treatment strategy to reduce the risk of bone metastasis. However, a hypermetabolic focus on left hemimandible was evidenced with a bone scintigraphy during follow up. After a careful study, the diagnosis of Bisphosphonate Related Ostneonecrosis of the Jaw (BRONJ) was carried out. This case highlights that bone scintigraphy may be extremely helpful for the early detection of BRONJ in high risk patient.

** Key words:**Bone scintigraphy, mandibular osteonecrosis, bisphosphonates.

## Introduction

Bisphosphonate Related Ostneonecrosis of the Jaw (BRONJ) associated with the use of intravenous bisphosphonates was described by Ruggiero in 2004 (1). Bisphosphonates work by modulating the following cellular activities: decrease of bone resorption due to inhibition of osteoclastic activity, induction of osteoclasts apoptosis, antiangiogenic action and alteration of the physiological bone turnover. Consequently, the bone becomes more fragile and its repair capacity is also reduced (2,3). In this context, the terminal vascularization and the poor soft tissue coverage of the maxilla facilitate the appearance of BRONJ. This complication may be serious and invalidating and undoubtedly represents a tough challenge for all oral and maxillofacial surgeons. Early diagnosis is essential to prevent severe sequels and to improve patient quality of life. The main objective of this paper is to report the utility of bone scintigraphy for the early detection of BRONJ.

## Case Report

We describe the case of a 62 years old woman underwent breast cancer surgery and axillar dissection in 2010 due to a ductal breast carcinoma (pT2N1M0). Patients received postoperative chemotherapy (Taxotere-Epirrubicine) and radiotherapy (50Gy) Intravenous bisphosphonates (Zometa ® 4mg monthly 4 per month) were also used to prevent bone metastasis, following this line.

**Figure 1:**
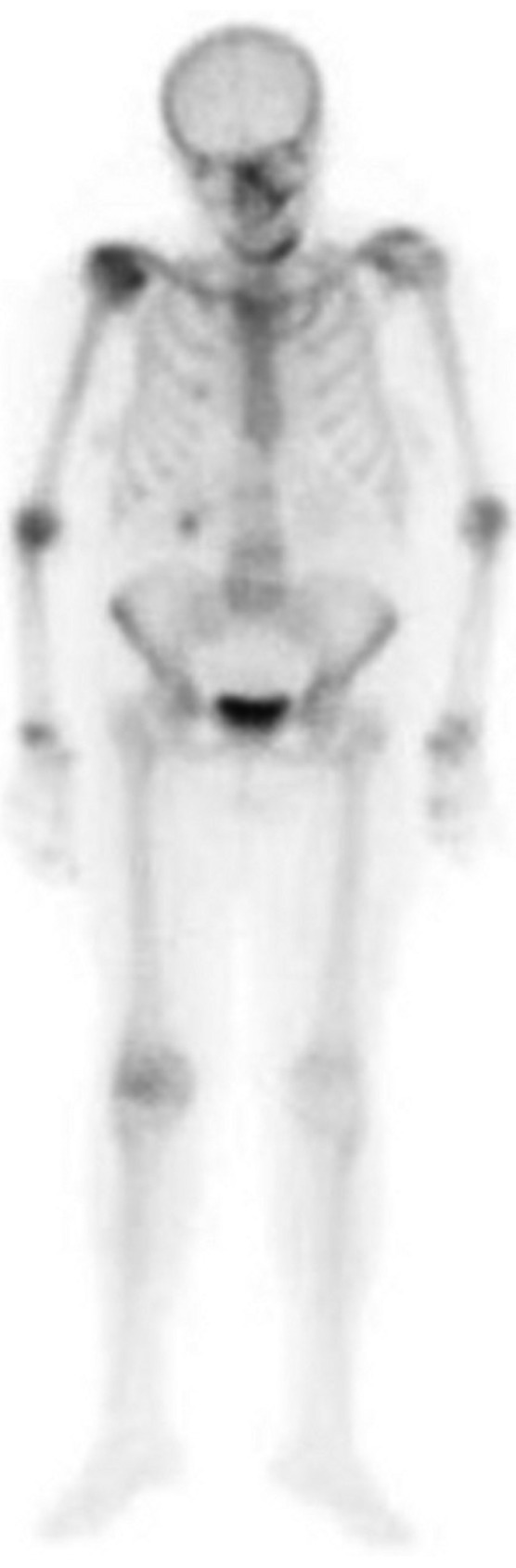
Bone scintigraphy that shows gadolinium enhancement. Enhancement on the right shoulder corresponds to chronic arthralgia. Enhancement on the left hemimandible is related to subclinical BRONJ.

**Figure 2:**
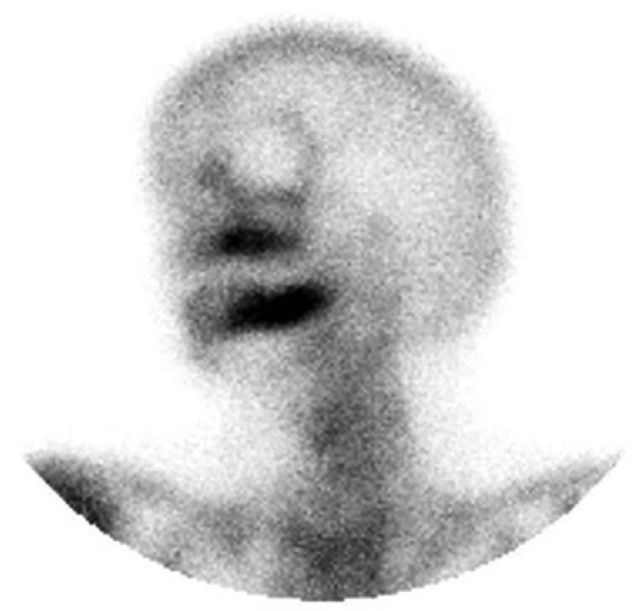
Pathological enhancement on left hemimandible (detail).

**Figure 3:**
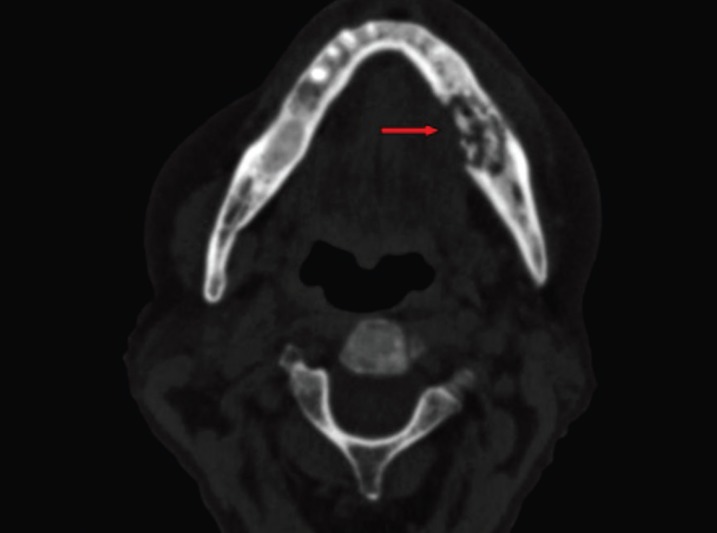
CT scan with osteosclerosis and bone sequestration areas, related to advanced stage BRONJ (red arrow).

A bone scintigraphy was performed two years after surgery as a routine control. Interestingly, hypermetabolic focus on right shoulder and left mandible were observed by this test. The increase in contrast uptake on the shoulder area was attributed to a chronic arthralgia. On the other hand, the evaluation of the focus involving left mandible was more difficult (Figs. 1,2). Intraoral examination did not show any significant finding, as well as the orthopantomography. However, an ulceration of the oral mucosa with clinical suppuration and bone exposition was observed three months later. A CT-scan showed radiological findings of BRONJ such as osteosclerosis and bone sequestration (Fig. 3). A biopsy finally confirmed the diagnosis of BRONJ. Patients was treated with surgical curettage of the area, soft tissue remodelling and medical treatment (oral amoxicillin/clavulanic acid 875/125 mg, 3 times a day, 15 days, associated with chlorhexidine 0.12% mouthwash 2-3 times a day).

Clinical evolution was favourable and patients and no recurrences were observed during follow up.

## Discussion

The uptake mechanisms of Bisphosphonates have not been completely clarified.

Several radiological test (functional, metabolic or morphologic) may be useful for the diagnosis of BRONJ (4). Bone scintigraphy is essential in assessing and classify patients affected by malignant tumours. Nowadays, it represents one of the most sensitive tests for bone metastasis detection. Moreover, it is extremely cost-effective. Technetium-99mm is the most commonly used medical radioisotope (5).

However, this test is not common in the early diagnosis of BRONJ and this represent the first case describing the clinical applications of bone scintigraphy for the early detection of this pathology. Orthopantomography, CT scan and MRI represent the tests most commonly used to diagnose BRONJ (6).

 However, several studies reported that CT scan might not show any significant findings at early stage (7,8) and it would not be useful for the early diagnosis of this pathology (9). In this sense, Mori et al remark the helpfulness of the MRI in the early diagnosis of asymptomatic BRONJ (10-13)

Other authors also proposed the use of other techniques, as Positron-Emission Tomography (PET) with fluorodeoxyglucosa. It could be performed associated with CT scan, perceiving early metabolic changes which are undetectable with simple clinical imaging (14).

The analysis of our case shows the fast evolution of BRONJ (2 years after the beginning of Zometa® treatment) in a patient treated with high power intravenous Bisphosphonates. This occur even in absence of precipitant risk factor is as reported by other authors (15). Hence, it is widely known the need of a close follow-up in the prevention of BRONJ.

In conclusion, this case suggests that functional changes in medulla and bone may precede the structural alterations detected with conventional tests. This highlights the potential need of developing a protocol for the early detection of BRONJ in high risk patients. In this scenario, bone scintigraphy may be extremely useful.
